# Three-week actigraphy to assess sleep behaviour and circadian rest-activity patterns in suspected and confirmed Cushing’s syndrome: an exploratory prospective cohort study

**DOI:** 10.1007/s11102-026-01686-6

**Published:** 2026-06-16

**Authors:** Annika Steinkogler, Stephanie Zopp, Adriana Albani, Marily Theodoropoulou, Isabel Stüfchen, Júnia R.O.L. Schweizer, Friederike Völter, Katrin Ritzel, Kathrin Moser, Katharina Schilbach, Martin Bidlingmaier, Martin Reincke, Elisabeth Nowak

**Affiliations:** 1 Department of Medicine IV, LMU University Hospital, LMU Munich, Ziemssenstrasse 5, 80336 Munich, Germany; 2 Department of Nuclear Medicine, LMU University Hospital, LMU Munich, Ziemssenstrasse 1, 80336 Munich, Germany; 3 Deggendorf Institute of Technology, Deggendorf, Germany

**Keywords:** Pituitary, Adrenal, Hypercortisolism, Chronotype, Late night salivary cortisol, ACTH, Wearables

## Abstract

**Purpose:**

Sleep disturbances are common in endogenous Cushing’s syndrome (CS), impair quality of life, and often persist despite remission. We evaluated the diagnostic value of actigraphy in suspected CS, characterised sleep and rest-activity patterns during remission, and examined associations between late-night salivary cortisol (LNSC) and objective sleep measures.

**Methods:**

Exploratory prospective single-centre cohort study at LMU Hospital Munich with two cohorts: (1) individuals evaluated for suspected CS and (2) patients in remission, stratified by recovered versus persistent adrenal insufficiency. Participants wore an ActTrust2 actigraph for 21 days, provided daily LNSC samples, and completed the Munich Chronotype Questionnaire.

**Results:**

Cohort 1 included 16 patients with confirmed CS and 13 with exclusion of CS. Actigraphy detected comparable sleep efficiency, fragmentation and circadian rest-activity rhythms in both groups, without distinct alterations attributable to active CS, except for a significantly earlier chronotype (p=0.022). Cohort 2 comprised 23 patients in remission (13 with persistent, 10 with recovered adrenal insufficiency) and showed similar sleep characteristics across subgroups. LNSC revealed high inter- and intraindividual variability without consistent associations with actigraphy-derived sleep parameters. Statistically significant effects were confined to patients with persistent adrenal insufficiency, in whom higher LNSC (likely reflecting non-physiological replacement therapy) was associated with earlier bedtime, longer time in bed, prolonged sleep-onset latency, and reduced sleep efficiency.

**Conclusion:**

Apart from chronotype, actigraphy showed limited discriminatory value between clinical groups. LNSC was highly variable and showed no consistent associations with sleep parameters, underscoring the need for improved biomarkers and monitoring strategies in CS.

## Introduction

Cortisol secretion follows a tightly regulated circadian rhythm, characterised by peak levels in the early morning and a nadir around midnight [1, 2]. In patients with endogenous Cushing’s syndrome (CS), chronic cortisol excess caused by ACTH- or cortisol-producing tumours disrupts this physiological rhythm, leading to persistently elevated cortisol concentrations that fail to decline at night [3]. This loss of rhythmicity contributes to the broad spectrum of cardiometabolic, infectious, musculoskeletal and neuropsychiatric complications that is characteristic for patients with CS and leads to increased morbidity and mortality [4].

Sleep disturbances and fatigue are among the earliest and most burdensome symptoms reported by patients before diagnosis, and fatigue remains the most frequently described persistent symptom after successful treatment, affecting up to 66% of patients [5]. Despite their clinical relevance, sleep disturbances and fatigue are addressed therapeutically in only ~ 16% of cases, according to a recent survey [5]. Patients with CS frequently experience insomnia, fragmented sleep, altered sleep architecture [6–8], and a markedly increased prevalence of obstructive sleep apnoea [9, 10]. Up to 69% report insomnia symptoms [11], and polysomnographic studies reveal reduced deep sleep, and altered rapid eye movement (REM) patterns [7, 12]. Almost 40% of treated patients continue to report poor sleep quality, and sleep medication use remains largely unchanged after remission [8]. Importantly, even less severe cortisol excess as observed in patients with mild autonomous cortisol secretion (MACS) is associated with substantial subjective sleep impairment [13]. Given the high prevalence and long-term persistence of sleep disturbances in CS, a better understanding of their characteristics and determinants is essential.

Wrist actigraphy provides a practical and scalable method for assessing sleep-wake and circadian rest-activity patterns in an outpatient setting over extended periods, allowing objective quantification of sleep duration, continuity, and fragmentation without the need for laboratory-based polysomnography [14]. Evidence in CS is limited, with only one actigraphic study in 12 patients demonstrating increased nocturnal motor activity and greater sleep fragmentation compared with healthy controls during a brief three-day recording period [15]. Nowadays, actigraphy studies typically employ longer observation periods of at least seven consecutive days and nights [16]. In clinical practice, a key diagnostic challenge is not distinguishing patients with overt CS from healthy individuals but differentiating them from patients referred for suspected hypercortisolism who share similar cardiometabolic and psychiatric comorbidities and may present with non-neoplastic hypercortisolism. Consequently, our study pursued three aims: (1) to evaluate the diagnostic utility of actigraphy in a prospectively enrolled cohort referred for suspected endogenous cortisol excess (i.e., with a high pre-test probability of having CS); (2) to characterise sleep architecture in patients with CS in long-term remission, including potential differences between individuals with and without adrenal insufficiency, thereby assessing the potential impact of (non-physiological) glucocorticoid replacement on sleep; and (3) to investigate associations between late-night salivary cortisol (LNSC) concentrations and actigraphic sleep parameters.

## Methods

### Study design and approval

This was an exploratory prospective monocentric observational cohort study at the LMU Hospital Munich conducted within the NeoExNet registry (Exzellenz-Netzwerk für Neuroendokrine Tumoren) between October 2023 and September 2024. The study was approved by the local ethics committee (study number: 152 − 10) and written informed consent was obtained from all participants. All investigations complied with the Declaration of Helsinki.

### Patient selection and clinical and biochemical evaluation of cortisol excess

This study included two distinct cohorts: cohort 1 included individuals prospectively evaluated for suspected endogenous glucocorticoid excess and cohort 2 included patients with CS in remission. Diagnosis and subtyping in both cohorts followed standardised clinical and biochemical protocols published previously [17, 18] according to current guidelines [19, 20]. In cohort 1, individuals without overt clinical signs of CS and with negative biochemical screening tests, were classified as having exclusion of glucocorticoid excess, and served as reference cohort. Patients with (MACS) [21] or with incomplete or inconsistent findings were excluded. Additional exclusion criteria for both cohorts were treatment with exogenous glucocorticoids (except replacement therapy after remission in cohort 2), active malignancy, and shift work. In cohort 2, remission was defined by prolonged adrenal insufficiency or adequate cortisol suppression (< 1.8 µg/dL) after a 1 mg dexamethasone test, following tumour resection and/or bilateral adrenalectomy. Patients in remission were classified as having recovered or persistent adrenal insufficiency, the latter receiving hydrocortisone acetate in two to three daily doses (maximum 25 mg/d).

### Questionnaire for assessment of chronotype and social jetlag

Chronotype and social jetlag were assessed using the Munich ChronoType Questionnaire (MCTQ) [22]. Chronotype refers to a biological construct reflecting an individual’s internal circadian timing rather than simple diurnal preference (“early bird” vs. “night owl”) [22]. It was estimated using the midpoint of sleep on work-free days, corrected for accumulated workweek sleep debt [22]. Social jetlag describes the misalignment between an individual’s biological circadian timing and socially imposed schedules (e.g., work times) [22]. It tends to be greater in later chronotypes, and has been associated with metabolic risk, unhealthy behaviors, and mood disturbances [23, 24]. It is quantified in the MCTQ as the difference between midsleep on workdays and work-free days [22]. To compare our findings, we included reference data derived from the Chronsulting platform (https://www.chronsulting.org/), a large dataset of healthy individuals with available MCTQ assessments. From this dataset, we selected an age- and sex-matched reference population that was ten times larger than our study cohort.

### Actigraphy for objective characterisation of sleep and rest-activity patterns

Wrist actigraphy was performed using the ActTrust 2 device (Condor Instruments, São Paulo, Brazil) for up to 21 consecutive days, including at least two full weekends to capture typical weekday-weekend variability. The ActTrust 2 monitors triaxial activity in 60-second intervals using a 3D accelerometer, ambient light, and temperature, enabling extraction of sleep onset latency, total sleep time, time in bed, wake after sleep onset, number of awakenings, and sleep efficiency. Participants wore the device continuously on the non-dominant wrist, removing it only when necessary (e.g., prolonged water exposure). They were instructed to press the event marker at bedtime and upon waking and recorded any deviations from their usual sleep-wake schedule to support accurate scoring of their sleep periods.

Actigraphy data were processed using the manufacturer’s software, and non-parametric circadian rhythm analysis was applied to quantify daily rest-activity patterns. Interdaily stability reflects the consistency of the 24-hour rhythm and ranges from 0 to 1, with higher values indicating greater day-to-day regularity. Intradaily variability quantifies rhythm fragmentation by capturing how frequently transitions occur between activity and rest or between light and dark; values approach 0 for smooth, sine-wave-like patterns and 2 for highly irregular, noise-like behaviour, with higher values indicating greater fragmentation. M10 represents the mean activity level during the 10 consecutive hours with the highest daily activity, while L5 represents the mean activity level during the 5 consecutive hours with the lowest activity. Relative amplitude, calculated as (M10-L5)/(M10 + L5), reflects the contrast between daytime and nighttime activity [25, 26]. The circadian function index combines several of these measures into a single score of overall circadian organisation. Finally, acrophase denotes the timing of peak daily activity and provides an estimate of circadian phase [27, 28].

### Prospective investigation of late-night salivary cortisol profiles

To investigate daily cortisol concentrations, all participants consecutively collected salivary samples using white Salivette^®^ tubes (Sarstedt, Germany) for LNSC measurement every evening before going to bed during the entire observation period of up to 21 days. Samples were obtained at least 30 min after food intake, tooth brushing, or smoking. Patients were instructed to freeze the saliva tubes in their local freezers at home (typical freezing temperature − 20 °C) until the end of their observation period. Saliva samples were subsequently thawed and measured at the endocrine laboratory at the LMU hospital Munich using an automated chemiluminescence immunoassay on the IDS iSYS analyser (Immunodiagnostic Systems, Boldon, UK). The analytical measuring range was 0.2–30 ng/mL, higher concentrations were diluted. The limit of quantification of the assay is 0.2 ng/mL. The intra-assay coefficient of variation and the inter-assay coefficient of variation are 5 and 9%, respectively. Assay performance characteristics have been validated by the manufacturer (IDS iSYS Salivary Cortisol ki, version 12.10.2022) and internally (https://doi.org/10.13140/RG.2.1.3482.0244). LNSC values were interpreted using an upper limit of normal ULN of 1.5 ng/mL.

### Statistical analysis

Statistical analyses were performed using GraphPad Prism version 10.1.1 and R version 4.2.2. Continuous variables were expressed as means with standard deviations (SD) for normally distributed data and as medians with interquartile ranges (IQR) for non-normally distributed data. Biochemical parameters were always reported as medians with IQR due to skewed distributions. Group differences were assessed using Welch’s t-test or the Mann-Whitney U test, as appropriate. Categorical variables were presented as percentages and analysed using Fisher’s exact test. Associations between LNSC and actigraphy-derived sleep parameters were assessed using mixed-effects models with a random intercept for patient ID to account for repeated measurements. LNSC values were log-transformed prior to analysis. All models were adjusted for age, BMI, and sex. Bedtime and wake-up timing were centered using group-specific circular means to account for the 24-hour periodicity of time and expressed as linear deviations (in minutes) from these means to improve interpretability. Continuous sleep outcomes (bedtime time, wake-up time, time in bed, total sleep time, sleep-onset latency, sleep efficiency, and wake after sleep onset) were analysed using linear mixed-effects models. The number of awakenings was analysed using generalised linear mixed-effects models with a log link and Poisson distribution; in case of overdispersion, a negative binomial model was applied. Effect estimates were presented as β coefficients for linear models and incidence rate ratios (IRR) for the count model, each with 95% confidence intervals. A two-sided P value < 0.05 was considered statistically significant.

## Results

### Characteristics of individuals evaluated for suspected endogenous cortisol excess (cohort 1)

In cohort 1, 40 patients with suspicion of endogenous hypercortisolism were consecutively enrolled for actigraphy and LNSC measurements. Among these, 16 (40%) were diagnosed with overt CS (9 pituitary, 6 adrenal, 1 ectopic). Fourteen individuals (35%) were found to have no evidence of endogenous cortisol excess; however, one of these was later excluded from analysis due to incompliance. Six patients (15%) with MACS, and four (10%) with an inconclusive diagnosis were excluded. Consequently, the final sample size for further analyses in cohort 1 comprised 16 patients with confirmed CS and 13 patients in whom CS was ruled out. Patients with CS had a mean age of 38.4 ± 9.1 years, were predominantly females (94%), and had frequent occurrence of typical cardiometabolic comorbidities. Patients in whom CS was excluded did not differ significantly in age, sex, BMI, or prevalence of comorbidities, but, by definition, showed significantly lower cortisol concentrations (Table 1).

**Table 1:** Baseline characteristics of cohort 1

	Active CS confirmed (*n* = 16)	CS ruled out (*n* = 13)	*p*-value
Demographic characteristics			
Age in years, mean ± SD	38.4 ± 9.1	44.4 ± 12.9	0.17
BMI in kg/m^2^, median (IQR)	28.5 (25.0-44.1)	29.9 (26.1–33.2)	0.98^†^
Female sex, n/N (%)	15 (94)	8 (62)	0.06
Comorbidities			
Diagnosed sleeping disorders, n/n (%)	0/16	0/13	1.0
Use of sleeping medication/tranquillizers, n/n (%)	1/16 (6)	0/13 (0)	1.0
Diagnosed obstructive sleep apnea, n/n (%)	2/16 (13)	0/13 (0)	0.49
Obesity, n/n (%)	8/16 (50)	6/13 (46)	1.0
Arterial hypertension, n/n (%)	8/16 (50)	8/13 (62)	0.71
Diabetes mellitus Type 2, n/n (%)	3/16 (19)	0/13 (0)	0.23
Pathological glucose tolerance, n/n (%)	5/16 (31)	1/13 (8)	0.16
Depression, n/n (%)	1/16 (17)	2/13 (15)	1.0
Biochemical evaluation			
Serum cortisol in µg/dL, median (IQR)	18.7 (12.6–22.4)	7.3 (4.4–13.4)	**0.0065** ^†^
Plasma ACTH in pg/mL, median (IQR)	49.5 (4.0–59.0)	12.0 (8.0–19.0)	0.32^†^
24 h-urinary free cortisol in µg/24 h, median (IQR)	362 (148–826)	80.3 (44.9–124)	**< 0.0001** ^†^
Median LNSC over 21 days in ng/mL, median (IQR)	9.1 (6.7–14.5)	1.5 (0.9–2.5)	**< 0.0001** ^†^
Response to 1 mg DST in µg/dL, median (IQR)	12.6 (6.1–15.5)	1.0 (0.7–1.3)	**< 0.0001**

Welchs t-test for normally distributed data, Mann-Whitney U test (†) for non-normally distributed data with p-values < 0.05 highlighted in bold. **Abbreviations: *ACTH* adrenocorticotropic hormone, *BMI* body mass index, *CS* Cushing’s syndrome, *DST* dexamethasone suppression test, *IQR* interquartile range, *LNSC* late night salivary cortisol

### Characteristics of individuals with CS in remission, with or without adrenal insufficiency (cohort 2)

In cohort 2, 25 patients were consecutively enrolled. Two patients were subsequently excluded due to relapse of CS. Among the remaining 23 patients, 13 (57%) had persistent adrenal insufficiency requiring glucocorticoid replacement, whereas 10 (43%) had recovered from adrenal insufficiency. Among patients with persistent adrenal insufficiency, seven had adrenal CS (five due to a unilateral adrenal adenoma treated by unilateral adrenalectomy, and two with bilateral micronodular adrenocortical disease treated by bilateral adrenalectomy), and six had Cushing’s disease (CD). All patients with CD had undergone transsphenoidal pituitary surgery, achieving remission in five, while one subsequently required bilateral adrenalectomy. Among patients with recovered adrenal insufficiency, seven had CD, all in remission after transsphenoidal pituitary surgery; two had adrenal CS due to a unilateral adrenal adenoma, both surgically cured; and one had ectopic CS caused by a neuroendocrine lung tumor that was successfully resected. Overall, clinical parameters and comorbidities were similar in both groups (Table 2).

**Table 2:** Baseline characteristics of cohort 2

	CS in remission, total (*n* = 23)	CS in remission with recovered adrenal insufficiency (*n* = 10)	CS in remission with persistent adrenal insufficiency (*n* = 13)	*p*-value
Demographic characteristics				
Age in years, mean ± SD	47.4 ± 13.4	52.0 ± 12.6	43.9 ± 13.3	0.15
BMI in kg/m^2^, median (IQR)	25.6 (23.7–31.1)	25.6 (24.0-30.1)	23.9 (23.6–31.8)	0.57^†^
Female sex, n/N (%)	16/23 (70)	6/10 (60)	10/13 (77)	0.65
Time since remission in months, median (IQR)	62.0 (24.0–86.0)	76.5 (41.3–121)	51.0 (12.0–73.0)	0.077^†^
Comorbidities				
Diagnosed sleeping disorders, n/n (%)	0/23 (0)	0/10 (0)	0/13 (0)	1.0
Use of sleeping medication/tranquillizers, n/n (%)	1/23 (4)	0/10 (0)	1/13 (8)	1.0
Diagnosed obstructive sleep apnea, n/n (%)	0/23 (0)	0/10 (0)	0/13 (0)	1.0
Obesity, n/n (%)	6/23 (26)	2/10 (20)	4/13 (31)	0.66
Arterial hypertension, n/n (%)	7/23 (30)	6/10 (60)	1/13 (8)	0.02
Diabetes mellitus Type 2, n/n (%)	2/23 (9)	2/10 (20)	0/13 (0)	0.18
Pathological glucose tolerance, n/n (%)	1/23 (4)	1/10 (10)	0/13 (0)	0.43
Depression, n/n (%)	2/23 (9)	0/10 (0)	2/13 (15)	0.49
Biochemical evaluation				
Serum cortisol in µg/dL, median (IQR)	-	5.1 (3.5–6.7)	0.6 (0.5–0.9)*	0.0004
Plasma ACTH in pg/mL, median (IQR)	-	18.0 (9.0–24.0)	9.5 (3.8–14.5)*	0.025
24 h-urinary free cortisol in µg/24 h, median (IQR)	-	94.8 (56.8–156)	-	*-*
Median LNSC over 21 days in ng/mL, median (IQR)	-	1.6 (1.1–2.5)	2.3 (1.3–4.7)**	**< 0.0001** ^†^
Response to 1 mg DST in µg/dL, median (IQR)	-	1.2 (0.9–1.3)	-	-

Welchs t-test for normally distributed data, Mann-Whitney U test (†) for non-normally distributed data with p-values < 0.05 highlighted in bold. Abbreviations: *ACTH* adrenocorticotropic hormone, *BMI* body mass index, *CS* Cushing’s syndrome, *DST* dexamethasone suppression test, *IQR *interquartile range, *LNSC* late night salivary cortisol. *Only in those without hydrocortisone intake on the day of sampling (*n* = 9). **Prospective saliva sampling only performed in 11 patients

### Evaluation of sleep parameters by actigraphy in cohort 1

We first compared sleep characteristics and rest-activity patterns in patients with confirmed CS vs. those in whom CS was excluded (cohort 1; Table 3). Both groups showed similar patterns regarding sleep efficiency, wake after sleep onset, and nocturnal awakenings. Likewise, circadian metrics (including interdaily stability, intradaily variability, relative amplitude, and activity during the most and least active periods) did not differ substantially, indicating comparable rest-activity rhythms in both groups. In contrast, both actigraphy and MCTQ showed significantly earlier chronotype in patients with CS than in those with exclusion of CS (*p* = 0.022 and *p* = 0.023, respectively). Importantly, chronotype in active CS was also significantly earlier than in age- and sex-matched healthy individuals derived from the Chronsulting database (active CS: 02:10 ± 00:41, healthy reference cohort: 03:43 ± 01:11, *p* = 0.0002). Social jetlag was slightly though not significantly greater in healthy individuals compared with active CS (active CS: 00:49 ± 00:27, healthy reference cohort: 1:16 ± 00:52, *p* = 0.068, Table 3). Taken together, chronotype was earlier in patients with active CS, whereas other sleep parameters where largely comparable between individuals with confirmed and excluded CS.

**Table 3:** Actigraphy in cohort 1

	Active CS confirmed (*n* = 16)	CS ruled out (*n* = 13)	*p*-value
Average wearing duration in days, mean ± SD	21.0 (21.0–21.0)	21.0 (20.0-22.5)	0.91^†^
Sleep characteristics by actigraphy			
Weekdays			
Bedtime in hh:mm	22:12 ± 1:28	22:39 ± 1:29	0.50^†^
Sleep-onset latency in minutes	00:53 ± 00:42	00:46 ± 00:19	0.85^†^
Wake up time in hh:mm	06:56 ± 01:17	06:50 ± 01:34	0.86
Awakenings in n	7.43 ± 3.19	8.29 ± 4.69	0.58
Sleep efficiency in %	79.7 ± 7.26	78.2 ± 5.25	0.52
Wake after sleep onset in minutes	00:39 ± 00:15	00:47 ± 00:29	0.91^†^
Total sleep duration in hh:mm	06:44 ± 00:31	06:18 ± 01:10	0.24
Total time in bed in hh:mm	08:35 ± 00:48	08:12 ± 01:14	0.33
Weekends			
Bedtime in hh:mm	22:18 ± 01:39	23:18 ± 01:28	0.095
Sleep-onset latency in minutes	00:53 ± 00:42	00:40 ± 00:27	0.50
Wake up time in hh:mm	07:22 ± 01:14	07:59 ± 01:44	0.29
Awakenings in n	7.10 ± 3.34	7.95 ± 3.78	0.53
Sleep efficiency in %	80.6 ± 7.63	80.0 ± 6.75	0.81
Wake after sleep onset in minutes	00:37 ± 00:17	00:43 ± 00:22	0.40
Total sleep duration in hh:mm	07:23 ± 01:09	06:43 ± 00:47	0.07
Total time in bed in hh:mm	09:21 ± 01:47	08:32 ± 01:01	0.13
Average activity over all days			
Onset of the most active 10 h (M10) in hh:mm	8:54 ± 2:38	8:46 ± 3:15	0.91
M10 average value in PIM	3604 ± 1139	3695 ± 1131	0.83
Onset of the least active 5 h (L5) in hh:mm	0:06 ± 1:19	0:20 ± 1:24	0.65
L5 average value in PIM	176 ± 71	395 ± 245	0.083^†^
Interdaily stability	0.5 ± 0.1	0.4 ± 0.1	0.38
Intradaily variability	0.8 ± 0.2	0.8 ± 0.2	0.90
Relative amplitude	0.9 ± 0.1	0.9 ± 0.1	0.20^†^
Circadian function index	0.6 ± 0.1	0.6 ± 0.1	0.35
Acrophase in hh:mm	13:42 ± 1:36	14:15 ± 1:30	0.35
Chronotype assessment			
Chronotype (MSF_sc_) through objective sleep timing through actimetry*	02:20 ± 00:58	03:41 ± 01:12	**0.022**
Chronotype (MSF_sc_) from MCTQ**	02:10 ± 00:41	04:01 ± 01:06	**0.023** ^†^
Social jetlag from MCTQ***	00:49 ± 00:27	01:30 ± 00:54	0.22

Welchs t-test for normally distributed data, Mann-Whitney U test (^†^) for non-normally distributed data with p-values < 0.05 highlighted in bold. *Chronotypes by actigraphy were only assessed in participants who did not use an alarm clock on weekends (CS = 10, CS ruled out = 8). **Chronotypes by MCTQ were only assessed in patients who did not use an alarm clock on weekends and who provided complete and fully analysable questionnaires (CS = 10, CS ruled out = 7). ***Social jetlag from MCTQ was only analysed in patients who provided complete and fully analysable questionnaires (CS = 12, CS ruled out = 10)

Reference MCTQ data from 160 age- and sex matched healthy individuals from the Chronsulting database, of whom 133 did not use an alarm clock on weekends: MSF_SC_ in healthy individuals = 3:43 ± 01:11 (CS *versus* healthy individuals: *p* = 0.0002; CS excluded *versus* healthy individuals: *p* = 0.47). Social jetlag in healthy individuals = 01:16 ± 00:52 (CS *versus* healthy individuals: *p* = 0.068; CS excluded *versus* healthy individuals: *p* = 0.85)

Abbreviations: *CS* Cushing’s syndrome, *MCTQ *Munich Chronotype Questionnaire, *MSF*_*sc*_ mid-sleep on free days, sleep corrected, *PIM *Proportional Integrating Measure

### Evaluation of sleep parameters by actigraphy in cohort 2

We then compared sleep characteristics and rest-activity patterns in patients with CS in remission with recovered vs. persistent adrenal insufficiency (cohort 2; Table 4). No significant differences in any sleep-related variable were detected between patients with recovered or persistent adrenal insufficiency (all *p* > 0.1). Sleep quality (including sleep efficiency, wake after sleep onset and nocturnal awakenings) was similar in both groups. Circadian activity patterns were likewise similar, with no relevant differences in the most active M10 (daily activity) or least active L5 period (nocturnal activity). Apart from a trend towards increased intradaily variability – indicative of fragmented 24-hour activity rhythms – in patients with recovered adrenal insufficiency vs. those with persistent adrenal insufficiency, which did not reach statistical significance (0.9 ± 0.2 vs. 0.7 ± 0.2, *p* = 0.081; Table 4), we did not observe significant differences in interdaily stability and intradaily variability. Patients with persistent adrenal insufficiency showed a trend towards earlier onset of the least active L5 period (*p* = 0.095) and an earlier acrophase, though not statistically significant (*p* = 0.079; Table 4). Chronotype and social jetlag were also similar between groups, and comparable to healthy individuals from the Chronsulting database. Together, these findings suggest that sleep and circadian measures are similar in patients in remission, regardless of adrenal recovery status.

**Table 4:** Actigraphy in cohort 2

	CS in remission, total	CS in remission with recovered adrenal insufficiency (*n* = 10)	CS in remission with persistent adrenal insufficiency (*n* = 13)	*p*-value
Average wearing duration in days, mean ± SD	21.0 (21.0–21.0)	21.0 (18.8–21.3)	21.0 (21.0–21.0)	0.56^†^
Sleep characteristics by actigraphy				
Weekdays				
Bedtime in hh:mm	22:30 ± 01:11	22:59 ± 01:22	22:08 ± 00:54	0.10
Sleep-onset latency in minutes	00:49 ± 00:33	00:51 ± 00:30	00:48 ± 00:36	0.84
Wake up time in hh:mm	07:26 ± 01:15	07:37 ± 01:11	07:18 ± 01:19	0.56
Awakenings in n	8.43 ± 3.71	8.23 ± 4.60	8.59 ± 3.06	0.83
Sleep efficiency in %	81.5 ± 6.36	80.9 ± 6.93	82.0 ± 6.13	0.70
Wake after sleep onset in minutes	00:39 ± 00:17	00:39 ± 00:18	00:40 ± 00:16	0.94
Total sleep duration in hh:mm	07:07 ± 00:45	06:51 ± 00:44	07:20 ± 00:44	0.14
Total time in bed in hh:mm	08:49 ± 00:54	08:33 ± 00:33	09:01 ± 01:06	0.17^†^
Weekends				
Bedtime in hh:mm	23:10 ± 01:16	23:34 ± 01:14	22:08 ± 00:54	0.10
Sleep-onset latency in minutes	00:50 ± 00:40	00:45 ± 00:27	00:48 ± 00:36	0.98^†^
Wake up time in hh:mm	08:07 ± 01:22	08:19 ± 01:11	07:58 ± 01:32	0.53
Awakenings in n	9.49 ± 4.16	8.73 ± 5.00	10.1 ± 4.41	0.83
Sleep efficiency in %	81.7 ± 7.97	81.5 ± 8.22	81.9 ± 8.10	0.70
Wake after sleep onset in minutes	00:41 ± 00:22	00:39 ± 00:19	00:44 ± 00:25	0.94
Total sleep duration in hh:mm	07:29 ± 00:54	07:12 ± 00:45	07:20 ± 00:44	0.14
Total time in bed in hh:mm	09:19 ± 01:15	09:00 ± 01:01	09:01 ± 01:06	0.19
Average activity over all days				
Onset of the most active 10 h (M10) in hh:mm	09:59 ± 2:21	10:30 ± 2:16	9:35 ± 2:35	0.48^†^
M10 average value in PIM	3741 ± 989	3382 ± 942	4018 ± 968	0.13
Onset of the least active 5 h (L5) in hh:mm	00:57 ± 00:54	01:19 ± 00:58	00:40 ± 00:45	0.095
L5 average value in PIM	188 ± 72	180 ± 55	193 ± 85	0.66
Interdaily stability	0.5 ± 0.1	0.5 ± 0.2	0.5 ± 0.1	0.64
Intradaily variability	0.8 ± 0.2	0.9 ± 0.2	0.7 ± 0.2	0.081
Relative amplitude	0.9 ± 0.05	0.9 ± 0.1	0.9 ± 0.05	0.41^†^
Circadian function index	0.7 ± 0.1	0.6 ± 0.1	0.7 ± 0.1	0.28
Acrophase in hh:mm	14:30 ± 1:13	15:02 ± 1:15	14:06 ± 1:04	0.079
Chronotype assessment				
Chronotype (MSF_sc_) through objective sleep timing through actimetry*	03:49 ± 01:04	03:50 ± 00:58	03:49 ± 01:13	0.97
Chronotype (MSF_sc_) from MCTQ**	03:28 ± 00:59	03:28 ± 01:21	03:18 ± 01:21	0.54^†^
Social jetlag from MCTQ***	01:18 ± 00:54	01:10 ± 00:51	04:24 ± 00:58	0.28^†^

Welchs t-test for normally distributed data, Mann-Whitney U test (^†^) for non-normally distributed data. *Chronotypes by actigraphy were only assessed in participants who did not use an alarm clock on weekends (recovered adrenal insufficiency = 7, persistent adrenal insufficiency = 8). **Chronotypes by MCTQ were only assessed in patients who did not use an alarm clock on weekends and who provided complete and fully analysable questionnaires (recovered adrenal insufficiency = 7, persistent adrenal insufficiency = 7). ***Social jetlag from MCTQ was only analysed in patients who provided complete and fully analysable questionnaires (recovered adrenal insufficiency = 7, persistent adrenal insufficiency = 8)

Reference MCTQ data from 230 age- and sex matched healthy individuals from the Chronsulting database, of whom 185 did not use an alarm clock on weekends: MSF_SC_ in healthy individuals = 3:33 ± 01:09 (total CS in remission *versus* healthy individuals: *p* = 0.86). Social jetlag in healthy individuals = 01:03 ± 00:44 (total CS in remission *versus* healthy individuals: *p* = 0.93)

Abbreviations: *CS* Cushing’s syndrome, *MCTQ* Munich Chronotype Questionnaire, *MSF*_*sc*_ mid-sleep on free days, sleep corrected, *PIM* Proportional Integrating Measure

### Association between prospective LNSC measurements and actigraphic data (cohorts 1 and 2)

**Fig. 1:**
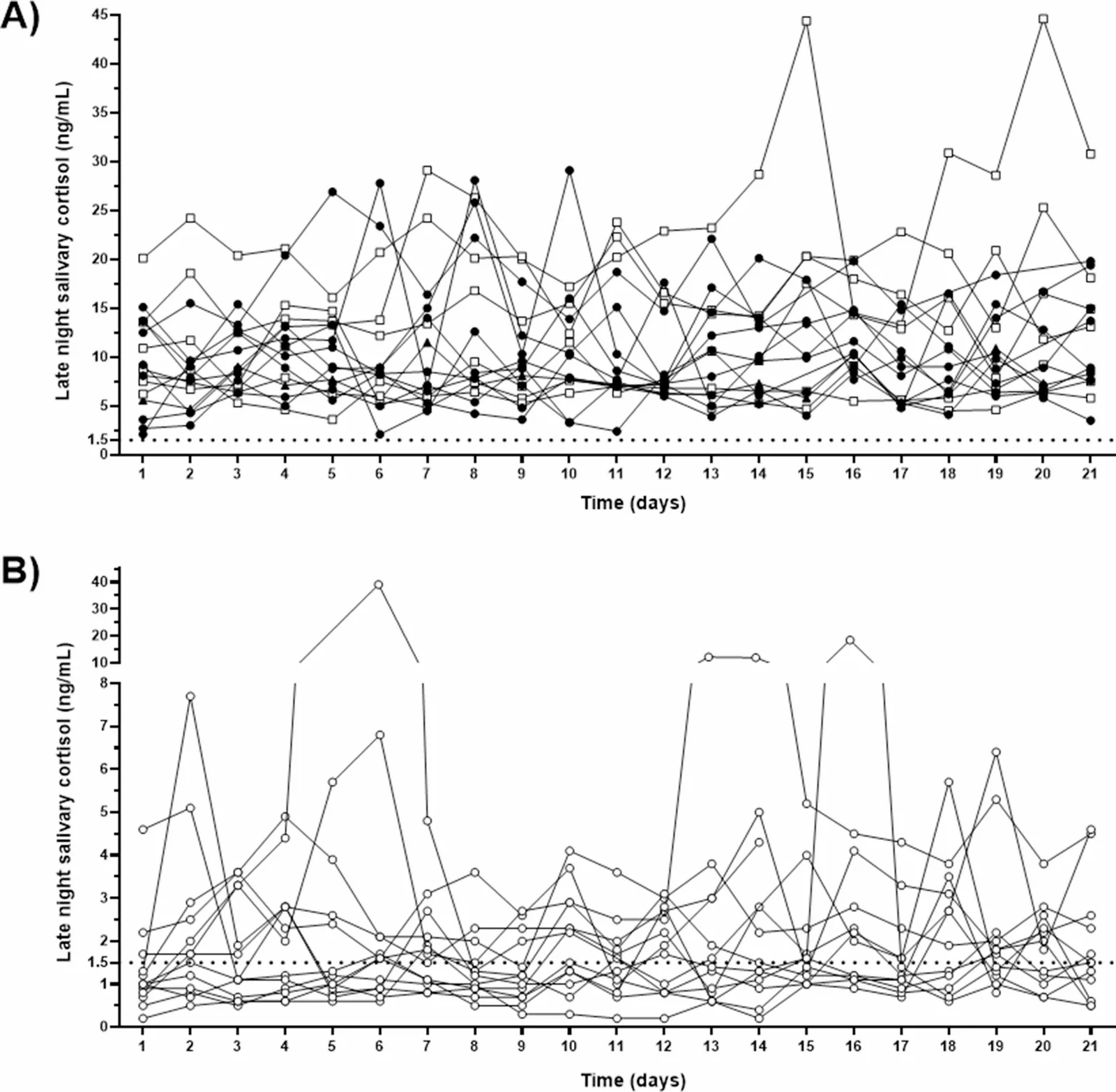
Prospective late-night salivary cortisol measurements in cohort 1. Horizontal dotted line indicates the upper limit of normal. **A**: Patients diagnosed with endogenous Cushing’s syndrome (CS); black circles, pituitary CS; white rectangles, adrenal CS; black triangles, ectopic CS. **B**: Individuals in whom CS was ruled out

**Fig. 2:**
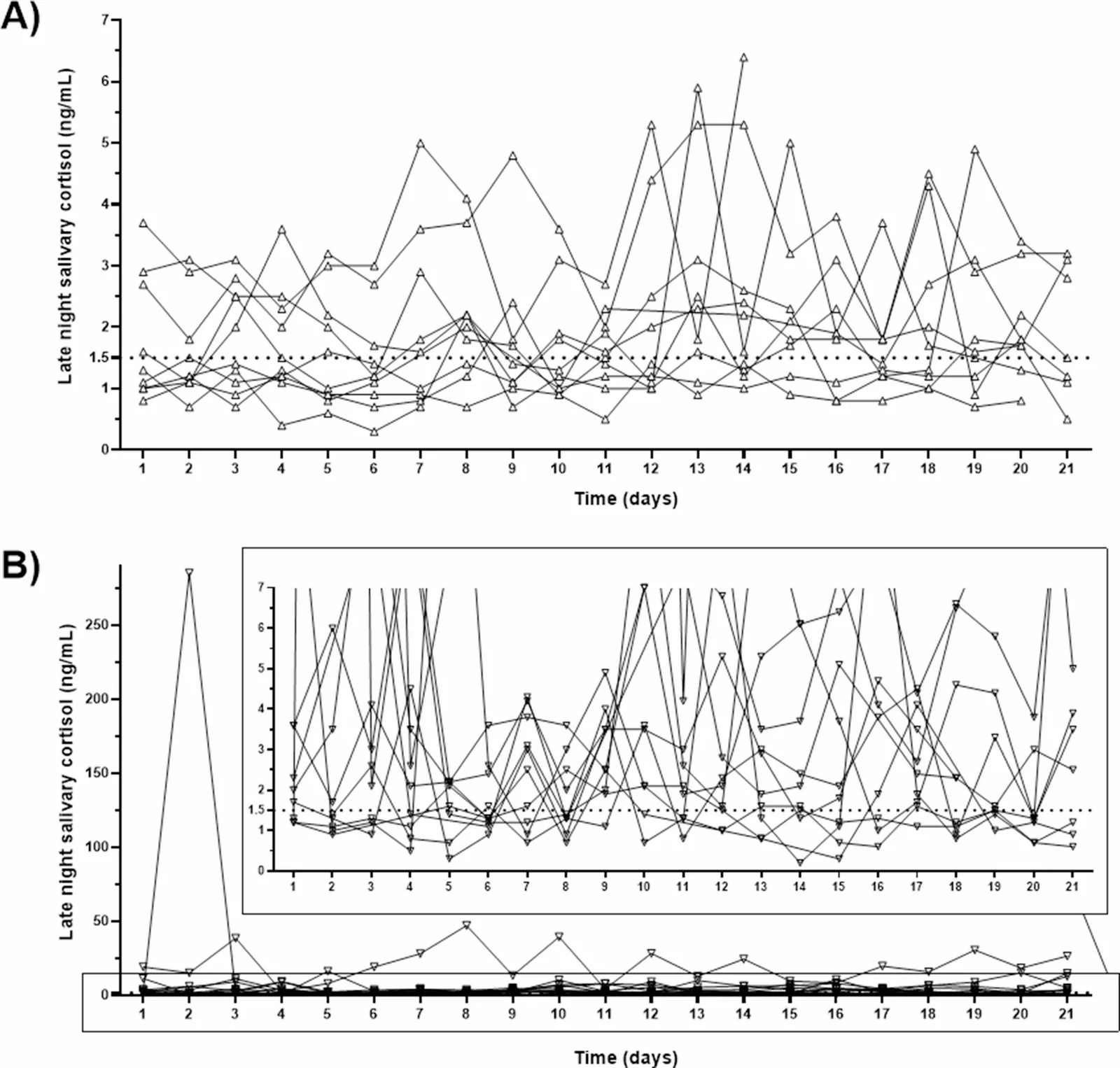
Prospective late-night salivary cortisol measurements in cohort 2. Horizontal dotted line indicates the upper limit of normal. **A**: Patients with Cushing’s syndrome in remission and recovery of adrenal insufficiency. **B**: Patients with Cushing’s syndrome in remission with persistent adrenal insufficiency requiring hydrocortisone replacement therapy. Cortisol concentrations reflect unphysiological replacement rather that endogenous cortisol production in these patients

**Fig. 3:**
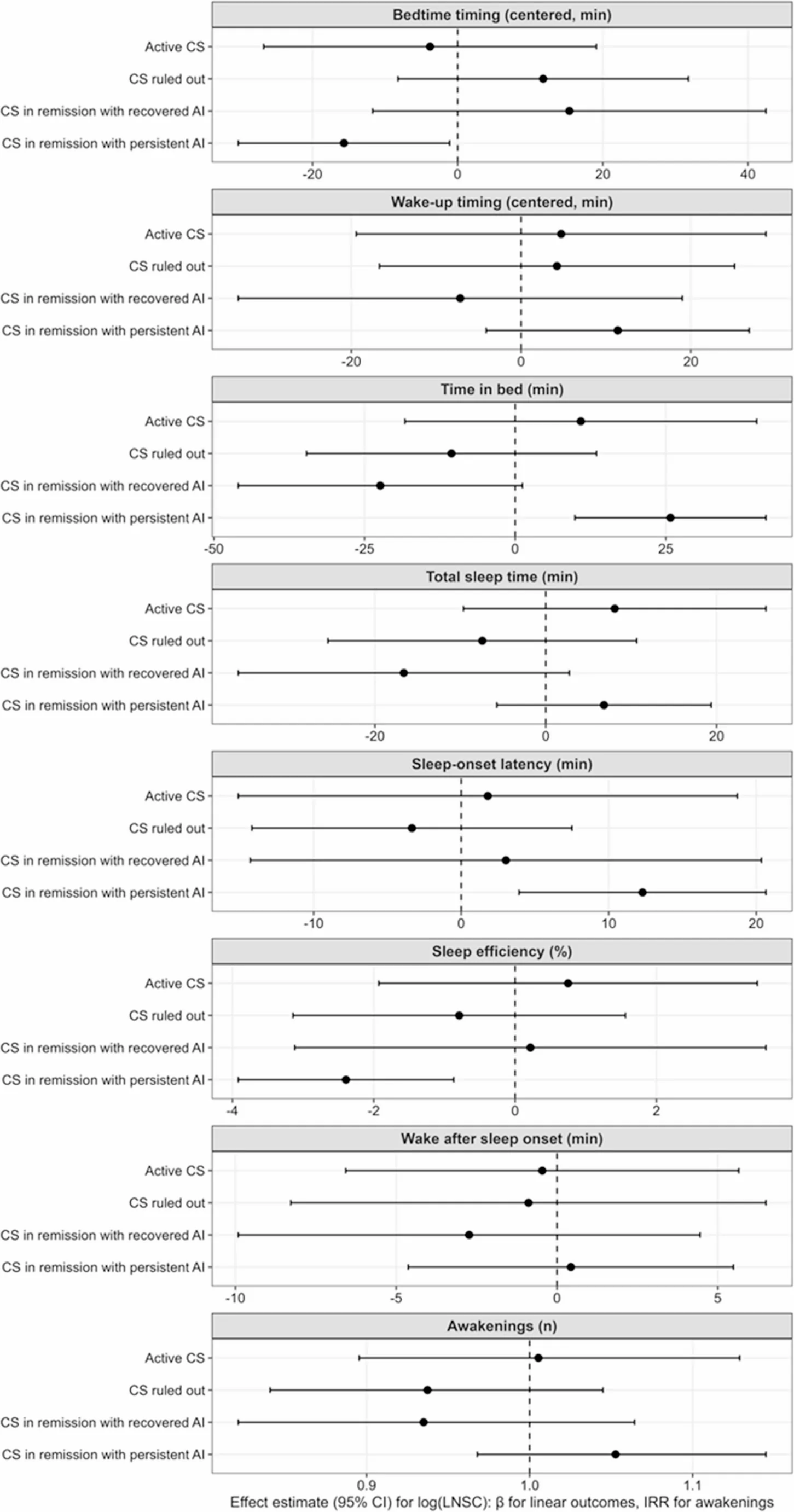
Association between log-transformed LNSC and sleep parameters. Forest plots show age-, sex- and BMI adjusted effect estimates across clinical groups. For continuous outcomes, points represent regression coefficients (β) with 95% confidence intervals from linear mixed-effects models. Bedtime and wake-up timing were circularly centered within each group (i.e., expressed as deviations in minutes from the group-specific mean). For awakenings, estimates are presented as incidence rate ratios (IRR) from count models. Abbreviations: AI, adrenal insufficiency; CS, Cushing’s syndrome; min, minutes; n, number

There was a high inter- and intraindividual variability in LNSC concentrations in patients with active CS, with 100% (330/330) of the measurements being above the ULN (Fig. 1A). We also observed considerable daily variation in patients with suspected but ruled-out CS, with 50% (132/263) of the samples being above the ULN (Fig. 1B). Of note, CS was reliably ruled-out in these patients by a combination of negative dexamethasone suppression tests and UFC, ACTH within the lower-medium reference ranges and lack of typical Cushingoid features. In cohort 2, marked variability was also present in remitted patients with recovery from adrenal insufficiency, in whom 55% (107/195) of LNSC samples were above the ULN (Fig. 2A). However, none of these patients was diagnosed with a relapse of CS, documented by repeatedly documented physiological suppression to dexamethasone and normal 24 h-UFC until more than one year after study completion (i.e., until December 2025). Patients with persistent adrenal insufficiency had mostly elevated LNSC (68%, 148/217), reflecting the unphysiological replacement regimen of hydrocortisone intake, rather than endogenous cortisol production (Fig. 2B). We next analysed if there were associations between LNSC concentrations and objective sleep parameters measured by actigraphy. Across groups, no consistent associations between log-transformed LNSC and most actigraphy-derived sleep parameters were observed in adjusted mixed-effects models. Statistically significant associations were identified only in patients with CS in remission with persistent adrenal insufficiency. In this group, higher LNSC was associated with earlier bedtime (relative to the group-specific mean), longer time in bed, longer sleep-onset latency, and reduced sleep efficiency. No statistically significant associations were observed for wake-up timing, total sleep time, wake after sleep onset, or nocturnal awakenings, nor in the other groups (Fig. 3).

## Discussion

In this prospective pilot study, we used three-week wrist actigraphy to characterise sleep and circadian rest-activity patterns in patients with suspected or confirmed CS and examined their relationship with LNSC. Actigraphy was feasible and yielded robust long-term data. Sleep and circadian parameters were broadly similar across the investigated groups (active CS, CS in remission, and patients in whom CS was ruled out), apart from a significantly earlier chronotype in patients with active CS. LNSC values were highly variable and overall showed limited correspondence with objective sleep measures.

Large normative polysomnography datasets in healthy adults typically report sleep efficiencies around 86–89% and lower nocturnal wake time, with only gradual age-related decline [29]. Across all groups in our study, sleep efficiency was approximately 80%, with prolonged nocturnal wake time and delayed sleep-onset. These findings indicate unfavourable sleep characteristics within our study population but cannot be interpreted as definitive impairment relative to a healthy reference group. The observed sleep patterns appeared largely independent of biochemical cortisol status and likely reflect a multifactorial interplay not only of hypercortisolism but also of psychiatric comorbidities, stress, and other non-endocrine contributors, as reflected by the comorbidity burden in our cohorts. Consequently, sleep complaints in this population warrant proactive clinical attention. Despite marked biochemical dysregulation in active CS, circadian rest-activity structure was largely comparable to individuals with ruled-out CS. This discrepancy is consistent with the concept of behavioural masking, whereby social routines, occupational schedules, and light exposure can obscure underlying circadian disturbances [30]. Because actigraphy captures behavioural output rather than endogenous hormonal rhythmicity, preserved activity rhythms do not exclude substantial internal circadian disruption. This notion is further supported by the substantially earlier chronotype observed in patients with active CS. The good agreement between actigraphy-derived and MCTQ-derived chronotype further supports the robustness of this circadian phase estimate in our cohort.

LNSC concentrations showed considerable intra- and interindividual variability, most pronounced in active CS but also present in individuals without CS and in patients in remission receiving hydrocortisone, in whom values primarily reflect replacement pharmacokinetics. This variability may be partly influenced by preanalytical factors, including incomplete adherence to sampling instructions, saliva contamination or dilution, and suboptimal sample storage or transport. Although the extreme oscillatory characteristics of cyclic CS with intermittent phases of spontaneous remission [31, 32] were not observed in our cohorts, substantial day-to-day fluctuations were evident and warrant clinical awareness. Importantly, patients in whom CS was excluded were not healthy controls but a clinically selected cohort referred for suspected hypercortisolism, which likely contributed to the higher proportion of LNSC values above the ULN. In patients with CS in remission and persistent adrenal insufficiency, higher LNSC levels were associated with a pattern of impaired sleep, characterised by longer sleep-onset latency and reduced sleep efficiency, alongside earlier bedtime and increased time in bed. Given that LNSC in this group likely reflects exogenous hydrocortisone exposure, these findings may suggest that higher evening glucocorticoid levels adversely affect sleep. The combination of earlier bedtime and prolonged time in bed, despite reduced sleep efficiency, may indicate compensatory behavior, potentially reflecting an attempt to counteract perceived insufficient or non-restorative sleep. In contrast, no statistically significant associations were observed in patients with active CS, despite endogenous cortisol excess, suggesting that LNSC alone may not adequately capture the aspects of cortisol dynamics relevant for sleep regulation in this context. Taken together, our findings underscore that sleep disturbances should be assessed clinically, and where appropriate, with direct sleep measures, as biochemical markers such as LNSC are insufficient proxies and may fail to capture unrecognised sleep disturbances. Moreover, awareness of pronounced day-to-day cortisol variability is essential, and repeated testing may be required to reliably detect or rule out endogenous CS.

Strengths of this study included the prospective design and the inclusion of patients with suspected but ultimately excluded CS as clinically meaningful comparators. Further strengths were the long actigraphy duration as well as rigorous diagnostic characterisation including daily LNSC measurements. However, several limitations should be acknowledged. The sample size was modest, reflecting both the rarity of the disease and the pilot nature of the study. Consequently, statistical power was limited, and negative findings should be interpreted with caution. Furthermore, no matched healthy control group was included. However, it was not our aim to reproduce previous findings [15] but to evaluate the diagnostic utility of actigraphy in patients with a high probability of having CS, which the study design adequately addressed. Accordingly, the findings describe sleep patterns within the studied cohort and should not be interpreted as definitive deviations from the general population. Interpretation of absolute sleep parameters is further limited by the absence of universally validated normative thresholds for actigraphy-derived metrics and the known inter-individual variability of sleep behaviour across age and lifestyle. At the same time, the availability of a large, matched healthy control population with MCTQ-based chronotype data represents a key strength, providing a robust external reference for circadian timing and supporting the validity of the observed associations. Finally, we acknowledge potential confounding by season affecting recorded light exposure. However, since participants in all subgroups were recruited throughout different seasons, any seasonal bias is likely to have affected all groups comparably.

In conclusion, the earlier chronotype observed in patients with active CS suggests a shift in internal circadian timing, even when behavioural rest-activity patterns appear similar. This subtle misalignment may contribute to typical patient-reported fatigue and sleep difficulties, but on its own is not sufficiently specific for diagnostic purposes. Actigraphy was useful for characterising habitual sleep behaviour and identifying clinically relevant sleep disturbances, but could not serve as an additional diagnostic tool for the detection of CS. Whether it has value for longitudinal monitoring in CS, such as during medical treatment or for early detection of relapse, remains uncertain and requires prospective investigation. Clinically, our findings translate into three key implications. First, sleep disturbances are frequent across all patients evaluated for CS – independent of biochemical status – and warrant systematic assessment and targeted management, including behavioural or medical interventions when appropriate. Second, LNSC is highly variable and should not be used as a proxy for sleep quality. Yet, in patients with persistent adrenal insufficiency, the potential impact of glucocorticoid replacement (particularly timing and dose) on sleep should be considered. Third, there remains a need for improved clinical monitoring strategies and more reliable biochemical biomarkers to aid the detection of active CS and its recurrence during follow-up. 

## Data Availability

The datasets generated and analysed during the current study are not publicly available but are available from the corresponding author on reasonable request.
